# Bridging Gaussian Density Fluctuations from Microscopic to Macroscopic Volumes: Applications to Non-Polar Solute Hydration Thermodynamics

**DOI:** 10.1021/acs.jpcb.1c04087

**Published:** 2021-07-20

**Authors:** Henry S. Ashbaugh, Mayank Vats, Shekhar Garde

**Affiliations:** † Department of Chemical and Biomolecular Engineering, Tulane University, New Orleans, Louisiana 70118, United States; ‡ Center for Biotechnology and Interdisciplinary Studies and the Howard P. Isermann Department of Chemical and Biological Engineering, Rensselaer Polytechnic Institute, Troy, New York 12180, United States

## Abstract

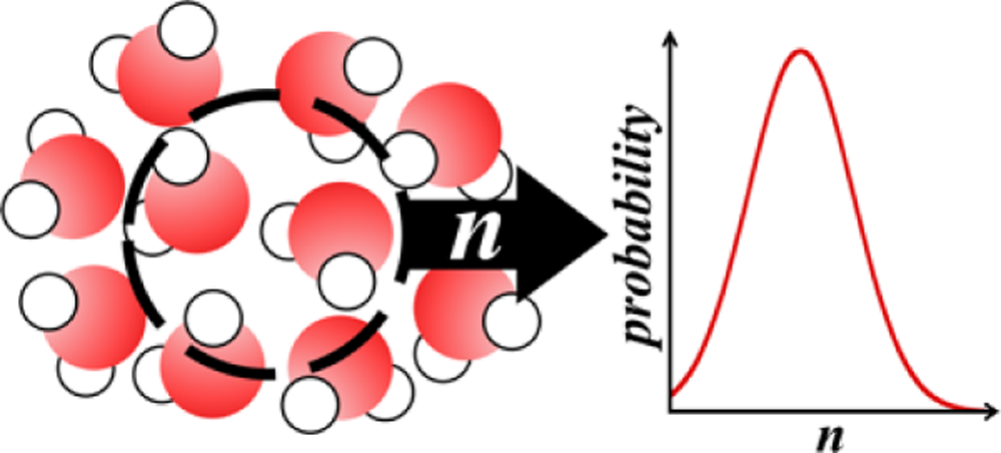

The hydration of hydrophobic solutes is intimately related to the spontaneous formation of cavities in water through ambient density fluctuations. Information theory-based modeling and simulations have shown that water density fluctuations in small volumes are approximately Gaussian. For limiting cases of microscopic and macroscopic volumes, water density fluctuations are known exactly and are rigorously related to the density and isothermal compressibility of water. Here, we develop a theory—interpolated gaussian fluctuation theory (IGFT)—that builds an analytical bridge to describe water density fluctuations from microscopic to molecular scales. This theory requires no detailed information about the water structure beyond the effective size of a water molecule and quantities that are readily obtained from water’s equation-of-state—namely, the density and compressibility. Using simulations, we show that IGFT provides a good description of density fluctuations near the mean, that is, it characterizes the variance of occupancy fluctuations over all solute sizes. Moreover, when combined with the information theory, IGFT reproduces the well-known signatures of hydrophobic hydration, such as entropy convergence and solubility minima, for atomic-scale solutes smaller than the crossover length scale beyond which the Gaussian assumption breaks down. We further show that near hydrophobic and hydrophilic self-assembled monolayer surfaces in contact with water, the normalized solvent density fluctuations within observation volumes depend similarly on size as observed in the bulk, suggesting the feasibility of a modified version of IGFT for interfacial systems. Our work highlights the utility of a density fluctuation-based approach toward understanding and quantifying the solvation of non-polar solutes in water and the forces that drive them toward surfaces with different hydrophobicities.

## Introduction

The dissolution of non-polar solutes in water and attractive forces between non-polar solutes mediated by water—so-called hydrophobic hydration and interactions—have been of interest for many reasons. An important motivation for studying such solvation phenomena is their relevance to many biophysical self-assembly processes,^1−3^ which has led to a long and rich history of experimental, theoretical, and simulation studies.^4,5^ In addition, the basic problem of dissolving a simple non-polar solute, for example, a hard-sphere, in water is in-and-of-itself interesting for it connects the molecular-level behavior of liquids—structure, density fluctuations, and so forth—to the thermodynamics of solvation. For example, the reversible work, μ_A_^ex^, to insert a hard-particle solute, A, in water is related to the probability, *p*_0_, of spontaneous formation of a cavity of the size and shape of the solute. An early work based upon the information theory^6^ viewed the formation of a cavity in a solute-sized observation volume, ν, as one element of the broader distribution *p*_*n*_, the probability of observing *n* water molecule centers within ν. Further, Hummer et al.^7,8^ showed that water density fluctuations in small volumes are nearly Gaussian and could be modeled easily from the knowledge of the average and the variance of *p*_*n*_, quantities directly related to the density and the radial distribution function of water, thus providing a route from two of the simplest measures of the water structure to the thermodynamics of hydrophobic solvation. This perspective focusing on water density fluctuations has been powerful and has provided insights into hydrophobic solvation near chemically diverse surfaces,^9^ proteins,^3^ and in other complex environments and has also motivated the development of new computational methods for the measurement of density fluctuations in larger volumes,^10−13^ which is not feasible in typical equilibrium molecular simulations. Those studies show that water density fluctuations in larger volumes (>1 nm radius) display increasingly non-Gaussian behavior in the low-*n* tails of the *p*_*n*_ distribution,^14,15^ indicative of the proximity of water under ambient conditions to its liquid-to-vapor phase transition and consistent with the corresponding signatures observed in the length-scale-dependent hydration of hydrophobic solutes and the associated crossover.^16^

For solutes smaller than the crossover length, where density fluctuations are effectively Gaussian, knowledge of the variance of *p*_*n*_ is particularly useful, for in conjunction with density, it enables the prediction of the excess chemical potential of hard particles in solution. In the information theory formalism, the variance is obtained from the integration of water’s radial distribution function, which in turn can be obtained either from simulations or scattering experiments. Here, we focus on the dependence of the variance on the size of select observation volumes in water. Given that the variance of solvent occupancy fluctuations in microscopic volumes (approaching zero size) and macroscopic volumes (approaching infinity) are known exactly from statistical mechanics, we explore the development of a new theoretical approach—interpolated gaussian fluctuation theory (IGFT)—to calculate the normalized variance over the entire solute size range from molecular to macroscopic. Our development is not unlike that of the scaled-particle theory,^17−19^ which smoothly interpolates between the known microscopic and macroscopic length-scale solvation behavior to build a smooth bridge over all solute sizes. We show that using only one parameter, the size of a water molecule, in combination with the knowledge of water density and compressibility, IGFT enables the prediction of the spherical solute chemical potential over a broad range of temperatures, reproducing the well-known entropy convergence in hydrophobic hydration for a range of solutes with sizes less than the crossover length scale. We also report data from molecular simulations to study the size dependence of the normalized variance in inhomogeneous systems containing self-assembled monolayers presenting hydrophobic and hydrophilic chemistries and discuss the challenges in developing an analytical interpolative theory to describe the interactions with extended surfaces. These simulations highlight interesting differences between hydrophobic solvation in bulk and interfacial environments. The approach described herein provides an alternate perspective for describing the hydration of non-polar entities that could be readily extended beyond aqueous systems. Moreover, IGFT illustrates that the water structure is not determinative of the characteristic thermodynamics of small-solute hydrophobic hydration, but rather they are embodied within the unique equation-of-state properties of water.

## Theory

The excess chemical potential of hydrating a hard-sphere (HS) solute (A), the contribution to the chemical potential above and beyond the ideal gas contribution, is determined by the probability of observing an empty cavity (*p*_0_) the same shape and volume of the solute within the bulk solvent as a result of ambient water density fluctuations
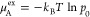
1For a HS solute with a solvent-excluded radius (*R*) less than half of the diameter of an individual water (w) molecule, *R* < *d*_ww_/2, at most one solvent molecule can fit within the bounds of a solute-sized observation volume. In this case, *p*_0_ = 1 – 4π*R*^3^ρ_w_/3, where ρ_w_ is the number density of water. Solutes with a water-excluded radius equal to half of water’s diameter, that is, *R* = *d*_ww_/2, correspond to point-like solutes with a hard-sphere radius equal to zero. However, to determine *p*_0_ for larger solutes, it is necessary to consider two-, three-, and higher-body water–water correlations, making the analytical determination of the aqueous solubility of realistically sized solute cavities increasingly difficult.

Given only water’s density and pair correlations, the information theory predicts water occupation probabilities within the solute-shaped observation volumes following the functional form^6^
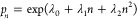
2Here, *p*_*n*_ is the probability that *n* solvent molecule centers are found within the observation volume, while the λ_*i*_’s are fitted to ensure the constraints
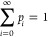
3a
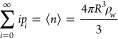
3band
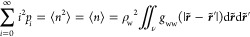
3care satisfied, where *g*_ww_(*r*) is the water oxygen–oxygen radial distribution function (RDF) and ν denotes the integration domain over the spherical cavity volume. These constraints ensure the probability is normalized and that its first and second moments match the observation. Despite neglecting three-body and higher-order correlations, eq 2 accurately captures the thermodynamics of hydrophobic hydration for atomic-sized solutes.^6,8^ Indeed, the inclusion of higher-order moments in the information theory description of *p*_*n*_ initially leads to worse predictions until the inclusion of seventh-order moments.^20^ Practical applications of the information theory to understand hydration have subsequently limited the constraints applied to only two-body correlations.

The information theory expression for *p*_*n*_ given above (eq 2) is a discrete analog to the Gaussian distribution. Assuming that eq 2 can be replaced by a continuous distribution, the solvent occupation fluctuations can be approximated as^7,8^
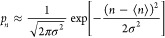
4where σ^2^ = ⟨*n*^2^⟩ – ⟨*n*⟩^2^ is the variance of the solvent occupation distribution. In addition to assuming that *n* is continuous, the normalization of this distribution assumes that *n* can be less than zero. The probability that *n* is negative, determined as *p*_*n*<0_ = ∫_–∞_^0^*p*_*i*_d*i*, is small and makes a negligible contribution toward the evaluation of the chemical potential of atomic-sized HS solutes and therefore is neglected here. Using eqs 4 and 1, the solute excess chemical potential is given by
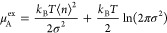
5To develop an analytical description of solute hydration, we recast this expression as
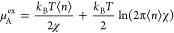
6where χ = σ^2^/⟨*n*⟩ = (⟨*n*^2^⟩ – ⟨*n*⟩^2^/⟨*n*⟩) is the normalized variance χ, which is determined by the integral of the water pair correlation function
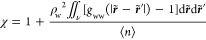
7This Gaussian framework has been shown to accurately describe solute hydration from point-like solutes up to those comparable in size to xenon. Beyond this scale, the Gaussian approximation gradually breaks down, especially in the low-*n* tail of the *p*_*n*_ distribution (which is key for determining *p*_0_!), reflecting the proximity of water under ambient condition to its liquid to vapor-phase transition and the associated crossover.^14,18,19,21,22^ Moreover, for a macroscopic solute, we expect the free energy to scale as the bulk pressure multiplied by the solute volume, while eq 6 predicts an effective pressure acting on a macroscopic surface of 1/(2κ_T_), where κ_T_ is water’s isothermal compressibility, which is ∼10,000 bar. While such effective pressures are reasonable on an atomic scale, it is unreasonable on the macroscopic scale. Similar overpressure corrections have been considered for integral equation and density functional theory predictions.^23−27^

Although the Gaussian framework accurately reproduces the hydration thermodynamics of atomic-scale solutes, χ must be obtained either by the evaluation of the integral in eq 7 using the known solvent RDF or by directly sampling density fluctuations from simulations. Either way, the solute size dependent χ is not known in the form of an analytical function. Progress on developing an analytical approximation for this thermodynamic variable, however, can be made by considering its limiting microscopic and macroscopic behavior. For a spherical observation volume, eq 7 can be re-expressed as
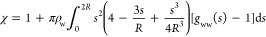
8For cavities smaller than the distance of closest approach between two water molecules, *d*_ww_, the pair correlation function is zero and eq 8 yields
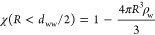
9In the limit of an infinitely large cavity, eq 8 effectively reduces to the Kirkwood-Buff^28^ integral for the compressibility yielding
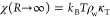
10Similar to the philosophical approach of the scaled-particle theory (SPT), which builds an interpolative formula for the free energy of hard solutes based on known limits,^17−19^ our goal then is to develop an expression that bridges χ from the known microscopic (eq 9) and macroscopic (eq 10) limits to describe solvent density fluctuations over all the length scales. Our focus on density fluctuations here will allow us to predict quantities such as cavity occupation distributions, which SPT does not address, although the range over which we can accurately predict hydration free energies is limited by the range over which the Gaussian approximation can be applied into the wings of the distribution. Considering a Laurent expansion of eq 8 in terms of 1/*R*, the first three derivatives in the limit of an infinite sphere (1/*R* → 0) are
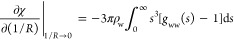
11a
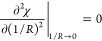
11band
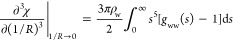
11cTherefore, while the first and third derivatives are determined by integrals over water’s RDF, the second derivative is identically zero. Like eq 8, the integrals in eqs 11a and 11c are not analytical, although they are expected to be finite away from the critical point. Considering the microscopic limit, χ and its first derivative are continuous at *R* = *d*_ww_/2. Based on these mathematical observations, we then propose that χ for cavities with *R* > *d*_ww_/2 can be expressed as a cubic polynomial in 1/*R*
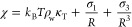
12Rather than evaluating eqs 11a and 11c explicitly, the coefficients σ_1_ and σ_3_ are fitted so that χ is smooth and continuous at *R* = *d*_ww_/2, while σ_2_ is zero as required by eq 11b. The resulting expression for χ over all the solute sizes is
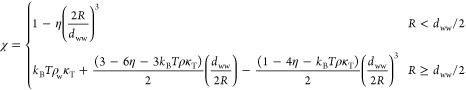
13where η = πρ_w_*d*_ww_3/6. The solvation free energy of a HS solute can subsequently be determined analytically by substituting eq 13 into eq 6. In addition, by evaluating the variance as σ^2^ =χ⟨*n*⟩, we can evaluate the cavity occupation probability distribution from eq 4. We refer to this approach as the IGFT. To the first approximation, IGFT finds that Gaussian occupation fluctuations within a spherical volume are captured by the macroscopic equation-of-state properties of water (density and compressibility) and a molecular length scale that describes the distance for which aqueous pair correlations begin to contribute to occupation fluctuations (*d*_ww_). Assuming *d*_ww_ is temperature-independent, eq 13 leaves *d*_ww_ as a single fitting parameter to describe HS solute hydration.

We note that philosophically IGFT is directly related to the extrapolation method proposed by Schnell and co-workers^29−32^ to evaluate Kirkwood-Buff integrals from simulations of finite, closed systems. In difference to that approach, however, IGFT utilizes the known compressibility evaluated from simulation fluctuations or experiment to construct a functional description of fluctuations over all the size scales.

### Molecular Simulations

#### Bulk Water Simulations

Molecular dynamics (MD) simulations of pure water were performed using GROMACS 5.^33^ Water was modeled using the TIP4P/2005 force field,^34^ which accurately captures water’s liquid equation-of-state properties. Non-bonded Lennard-Jones interactions were truncated beyond a separation of 9 Å with a mean-field dispersion correction for longer-range contributions to the energy and pressure. Electrostatic interactions were evaluated using the particle mesh Ewald Summation method with a real space cutoff of 9 Å.^35^ Simulations of 909 water molecules were performed at temperatures ranging from −20 to 325 °C in 5 °C increments at a pressure of 300 bar. This elevated pressure was used to ensure water remained a liquid at all temperatures. The temperature and pressure were regulated using the Nosé–Hoover thermostat^36,37^ and the Parrinello–Rahman barostat,^38^ respectively. Water was held rigid using the SETTLE algorithm.^39^ Following 2 ns of equilibration, each state point was simulated for 200 ns for the evaluation of equilibrium averages. The equations of motion were integrated using a time step of 2 fs. Simulation configurations were saved every 1 ps (200,000 total configurations at each state point) for post-simulation analysis of thermodynamic averages.

Mean and mean-square solvent number observation volume occupation averages were evaluated by randomly inserting spherical observation volumes within each solvent configuration. Spherical observation volumes up to 15 Å in radius were considered. Fluctuation averages were evaluated by performing 2000 random insertions in each solvent configuration.

The probability of observing *i* water oxygens in a spherical observation volume of radius *R* was evaluated following Widom’s test particle insertion formula in the isothermal-–isobaric ensemble^40,41^
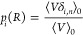
14In this expression, *n* is the instantaneous number of water’s in the observation volume, δ_*i*,*n*_ is the Kronecker delta, *V* is the volume of the simulation box, and the angle brackets ⟨...⟩0 indicate the averages performed over pure solvent configurations. Averages were conducted by performing 12,000 random insertions in each saved solvent configuration. We note that by performing simulations at 300 bar, the solute hydration-free energies observed will be approximately *P*ν̅_A_ greater than at atmospheric pressure, where ν̅_A_ is the solute’s partial molar volume. This perturbation, however, is less than *k*_B_*T* for the largest solute considered (*R* = 3.6 Å) over the entire simulated temperature range. We therefore expect the free energies determined here to be a reasonable representation of those that would be evaluated at coexistence.

#### Inhomogeneous System Simulations

We simulated model self-assembled monolayer (SAM) surfaces using a setup similar to that previously described by Garde and co-workers.^42,43^ A single SAM leaflet was prepared using 528 surfactant chains, each chain comprising 10 carbon atoms attached to a sulfur atom at the base and capped at the top with −OH or −CH_3_ head groups leading to a homogeneous hydrophilic or hydrophobic surface, respectively. The alkyl thiol chains of the SAM strands were modeled as united atoms,^44^ while the −OH and −CH_3_ head groups were parameterized using the generalized AMBER force field^45,46^ and AM1-BCC charges^47^ derived from methanol and ethane, respectively. The sulfur atom of each chain as well as the seventh carbon atom from the sulfur were fixed in their respective locations using a harmonic potential of 1000 kcal/(mol Å^2^), as previously done. The positions and orientations of the SAM chains correspond to an alkyl thiol SAM immobilized on a gold (111) surface.^48^ Water was modeled using the TIP3P potential.^49^ A periodic box size of 109.78 Å × 103.68 Å × 100 Å included the SAM leaflet solvated with ∼94,000 water molecules (−CH_3_ surface—94,503 waters; OH surface—93,858 waters). MD simulations were performed using GROMACS 2019.4^33^ in the isothermal–isobaric ensemble. The Nosé–Hoover thermostat^36,37^ and the Parrinello–Rahman barostat^38^ were used to maintain a temperature and pressure of 25 °C and 1 bar, respectively. Electrostatic interactions were calculated using particle-mesh Ewald summation.^35^ A cutoff distance of 9 Å was used for non-bonded interactions. Bonds containing hydrogens were constrained using the LINCS algorithm.^50^ Simulations were run for 10 ns with a time-step of 2 fs, saving configurations every 2 ps. The first 200 ps were used for equilibration, while the final 9.8 ns were analyzed to obtain simulation averages over a total of 4900 configurations.

While the TIP3P model used to examine SAM interfaces is distinct from the TIP4P/2005 model used in our bulk simulations, water density fluctuations near interfaces are expected to be comparable for the two models. The simulations of TIP3P water had been conducted prior to the development of IGFT. Here, we reanalyzed those interfacial simulations using TIP3P water. The trends observed and conclusions drawn, however, should not depend significantly on the water model used.

## Results and Discussion

### Water’s Equation-of-State and Fluctuations in the Bulk and at Surfaces

**Figure 1:**
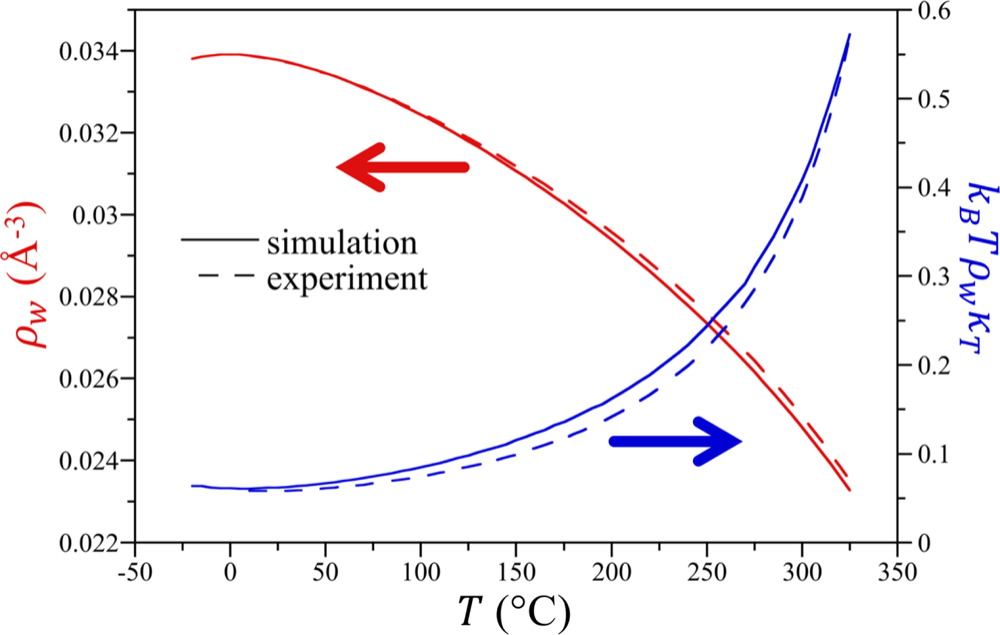
Comparison between the simulation and experimental number densities and normalized compressibility of water as a function of temperature from −20 to 300 °C at 300 bar. The simulation results are for TIP4P/2005 water. The experimental results, reported only from 0 to 325 °C, were obtained from the NIST Chemistry WebBook.^51^ The figure symbols are defined in the figure legend. The arrows indicate the corresponding *y*-axis for the density and compressibility data sets. Simulation error bars are smaller than the line thicknesses.

The density and normalized compressibility (*k*_B_*T*ρ_w_κ_T_) of TIP4P/2005 water as determined by simulation are reported as a function of temperature from −20 °C to 325 °C at 300 bar in Figure 1, which are used as inputs to IGFT (eq 13). These results are in excellent agreement with those from the experiment (reported from 0 to 325 °C at 300 bar obtained from the NIST Chemistry WebBook^51^), giving confidence that our simulations provide an accurate description of water’s equation-of-state over the state points considered. It is interesting to note that while the experiments do not exhibit a temperature of maximum density (*T*_md_) at this elevated pressure, the simulations predict a *T*_md_ in the supercooled regime at −0.4 ± 0.2 °C. This follows from the expectation that the *T*_md_ will drop below the freezing point with increasing pressure.^52^

**Figure 2:**
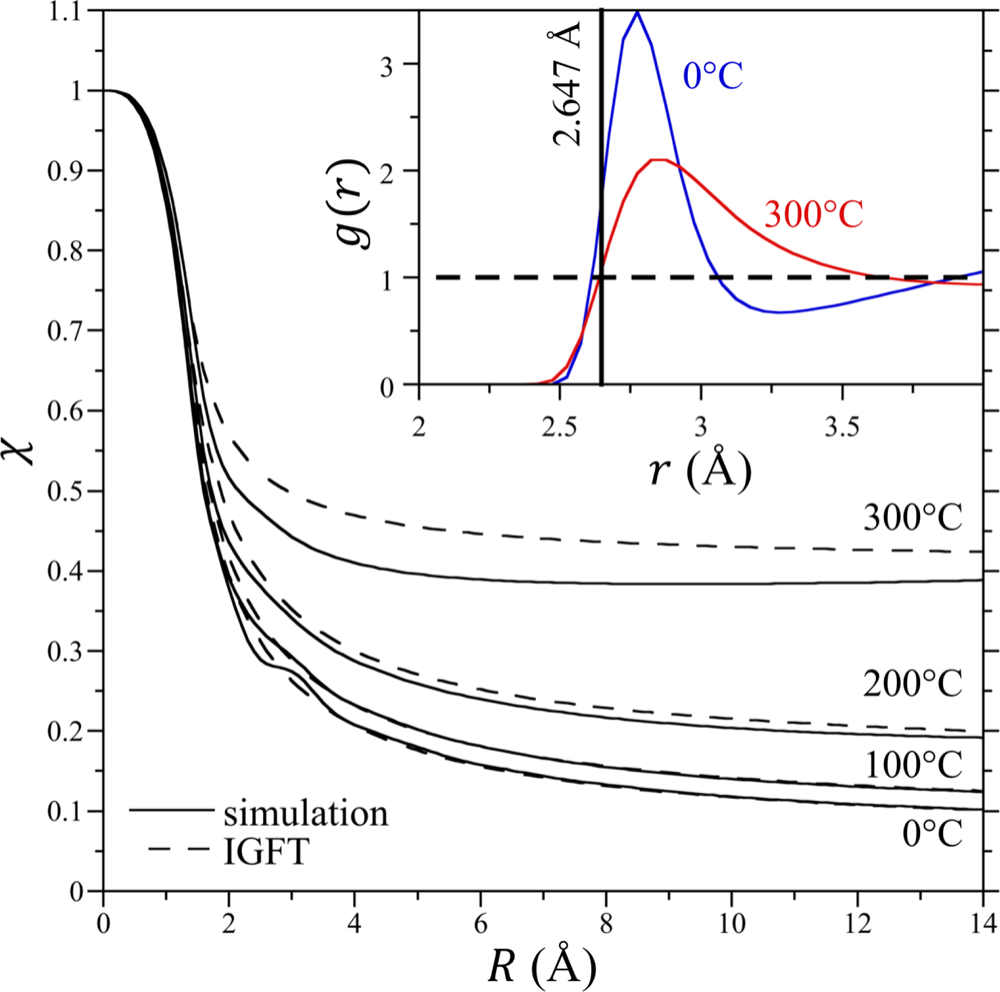
Normalized occupancy fluctuations as a function of the cavity radius in TIP4P/2005 water. The main figure reports the simulation and theoretical results (eq 13) for χ as a function of the cavity radius at 0, 100, 200, and 300 °C (temperatures identified in the figure) and 300 bar. The symbols are defined in the figure legend. Simulation error bars are smaller than the figure symbols. The inset figure shows details of the water oxygen–oxygen radial distribution function in the neighborhood over which it first crosses one at 0 and 300 °C. The RDF curves are identified in the figure. The vertical black solid line corresponds to *d*_ww_ = 2.647 Å, while the horizontal dashed line corresponds to a value of one.

The normalized water occupation variance, χ, in spherical observation volumes in bulk TIP4P/2005 water as a function of *R* at 0, 100, 200, and 300 °C at 300 bar determined from simulation is reported in Figure 2. Beginning at zero radius, χ determined from simulation is one and decreases with increasing solute size. Over the temperature range reported in this figure, χ is an increasing function of temperature for all cavity sizes. For observation volumes comparable in size to an individual water molecule (∼3 Å), a slight oscillation in χ is observed in the simulation results, attributable to the packing of water molecules in their mutual hydration shells on this length scale. This oscillation is most prominent at 0 °C and diminishes with increasing temperature as a result of the reduction of the first packing peak in water’s oxygen–oxygen RDF (Figure 2 inset). Beyond this length scale, χ decreases monotonically with increasing observation volume size at 0, 100, and 200 °C, approaching the macroscopic value of *k*_B_*T*ρ_w_κ_T_ for an infinitely sized volume. At 300 °C, however, a shallow minimum in χ is observed at ∼9 Å from the simulation after which χ monotonically increases toward its macroscopic limit.

The IGFT predictions for χ in bulk water (eq 13) are compared with simulation data in Figure 2 using a fitted *d*_ww_ of 2.647 Å. While this diameter is slightly smaller than that typically used for water ranging from 2.7 to 2.8 Å, this length corresponds approximately to the distance where the water oxygen–oxygen RDF first crosses one for the first time (Figure 2 inset). We may then conclude that this length corresponds to the point for which water–water pair correlations begin to contribute to the fluctuation integral (eq 8). Overall, IGFT provides an excellent description of χ, especially at 0–200 °C. Given that IGFT includes only information about the size of a water molecule, water density, and compressibility, it does not capture the packing oscillation near *R* ∼ 3 Å. Nevertheless, the theory threads the simulation results reasonably well. At 300 °C, however, the theory fails to capture the minimum in χ observed from simulation. The theory does exhibit a minimum in χ if a larger value of *d*_ww_ (on the order of 3 Å) is used; however, this reduces the theory’s predictive utility if *d*_ww_ is assumed to be a temperature-dependent parameter. For simplicity, we then accept the errors in the predicted values of χ and examine the consequences of assuming *d*_ww_ as temperature-independent below.

**Figure 3:**
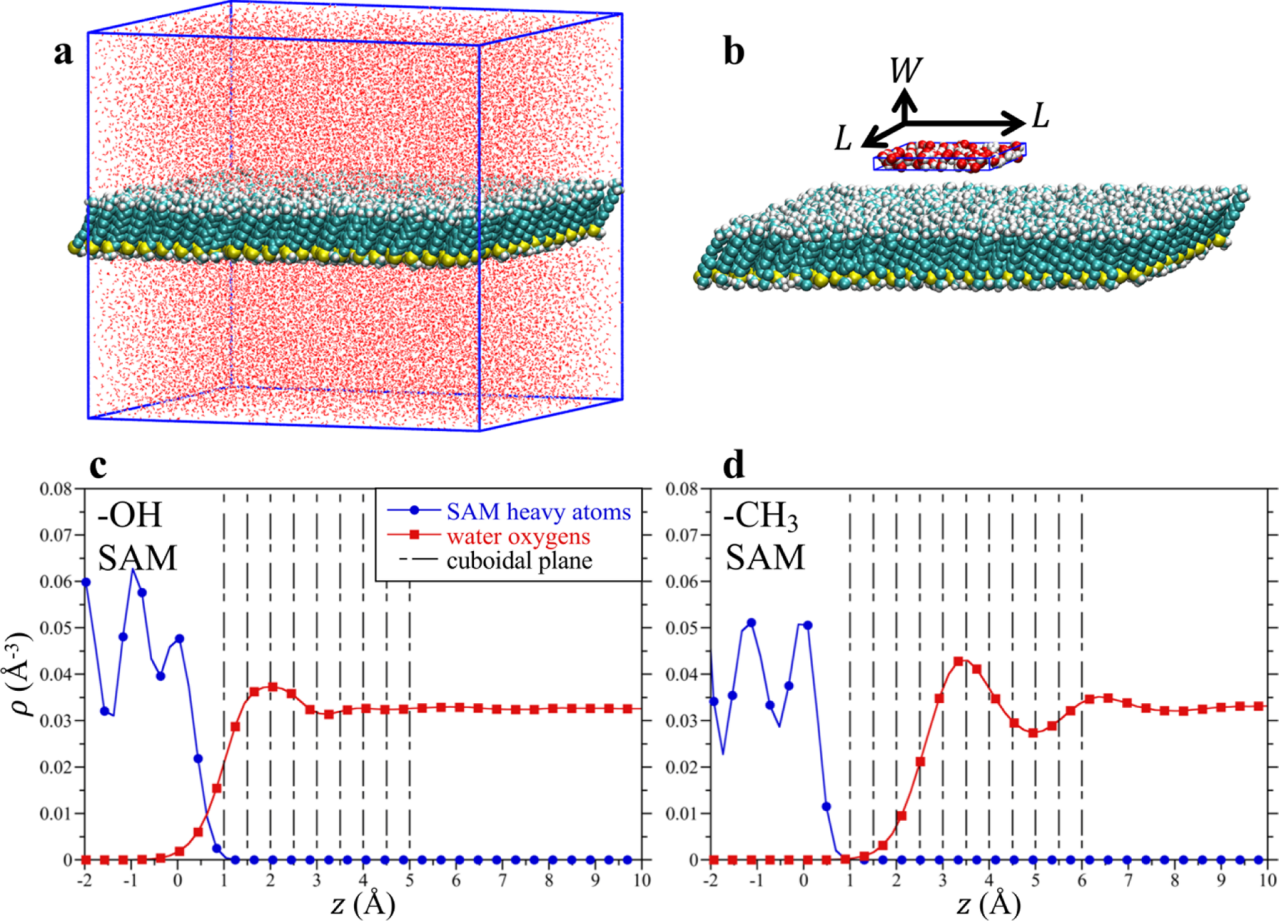
(a) Snapshot of the −CH_3_ SAM–water system in a 3D periodic box obtained from an MD simulation trajectory. A slab of SAM (carbons—cyan, hydrogens—white, and sulfur—yellow) is shown as hydrated in water (shown with a stick model). (b) Schematic of a *L* × *L* × 3 Å^3^ cuboid placed approximately 16 Å above the −CH_3_ SAM surface. Water molecules within the cuboid are shown using the space fill representation (oxygens—red and hydrogens—white). (c,d) Number density profiles for the heavy atoms of the −OH and −CH_3_ SAMs, respectively, as well as the water oxygen number density profiles for those two systems. For simplicity, we place the origin of the *z*-axis to coincide with the first peak of the SAM heavy atom density profile. (c,d) also show the locations of the central plane of the cuboid in both systems, sampling the region near the surface (shown with 9 and 11 vertical lines that are 0.5 Å apart, in −OH and −CH_3_ systems, respectively) as well as three locations in the bulk at distances of 10, 15, and 20 Å from the surface (not shown in figure).

When the observation volume is present near an interface, the nature of aqueous fluctuations in its vicinity is expected to depend on the proximity of the observation volume and the chemical composition of that interface. Figure 3 shows snapshots of a SAM terminated with the hydrophobic −CH_3_ head groups in contact with slabs of liquid water and highlights a representative cuboid placed *Z* Å away from the surface. While we have discussed the hydration of SAM surfaces in detail elsewhere,^9^ we highlight some of the more salient features that differentiate water at these surfaces from the bulk. Water molecules display layering near both the −OH- and −CH_3_-terminated SAMs. The local density of water as characterized by the first peak of the water density distribution along the *z*-axis normal to the SAM, however, does not correlate with the hydrophobicity or -philicity of the SAM. Notably, the first peak near the hydrophilic surface is smaller than that next to the hydrophobic surface. Moreover, the secondary peak is almost non-existent near the hydrophilic surface, while the hydrophobic surface exhibits a stronger secondary peak, indicative of more prominent layering of water at the interface. Cues to the relative favorability of hydration of these two surfaces are evident in the overlap between the density distribution of the SAM units and those of water. Specifically, the water density overlaps significantly with those of the −OH-terminated surface (suggesting the intercalation of water with the SAM head groups), while there is a nearly 2 Å gap between the water and −CH_3_-terminated surface-density distributions. The width of the gap between the water and SAM layers in turn has been shown to correlate with the surface contact angle,^9^ with the gap being wider for more hydrophobic surfaces. As pointed out by Godawat et al*.*, this gap—the so called “width of the interface”—is smaller than the size of a water molecule, and it is neither practical to measure it nor use it as a measure of the local hydrophobicity of a surface, especially that of a protein. Significant work over the past decade has clarified the underlying physics of hydrophobic surface hydration. Water dewets from an idealized hard-wall surface forming a molecularly thick vapor layer near the wall. Unlike that in the bulk, however, the density in this region is highly sensitive to perturbations (e.g., small amount of attractions with the surface). Realistic surfaces, such as the −CH_3_-terminated surface, exert sufficient van der Waals attractions pinning the liquid phase close to it, leading to an apparent liquid-like local density and the layering of water. The tendency of water to dewet the surface, however, remains and is evident not in the average but in the fluctuations of water density and associated quantities. Below, we explore how those density fluctuations manifest themselves in the measurements of χ in cuboidal volumes of increasing sizes near −CH_3_ and −OH surfaces.

**Figure 4:**
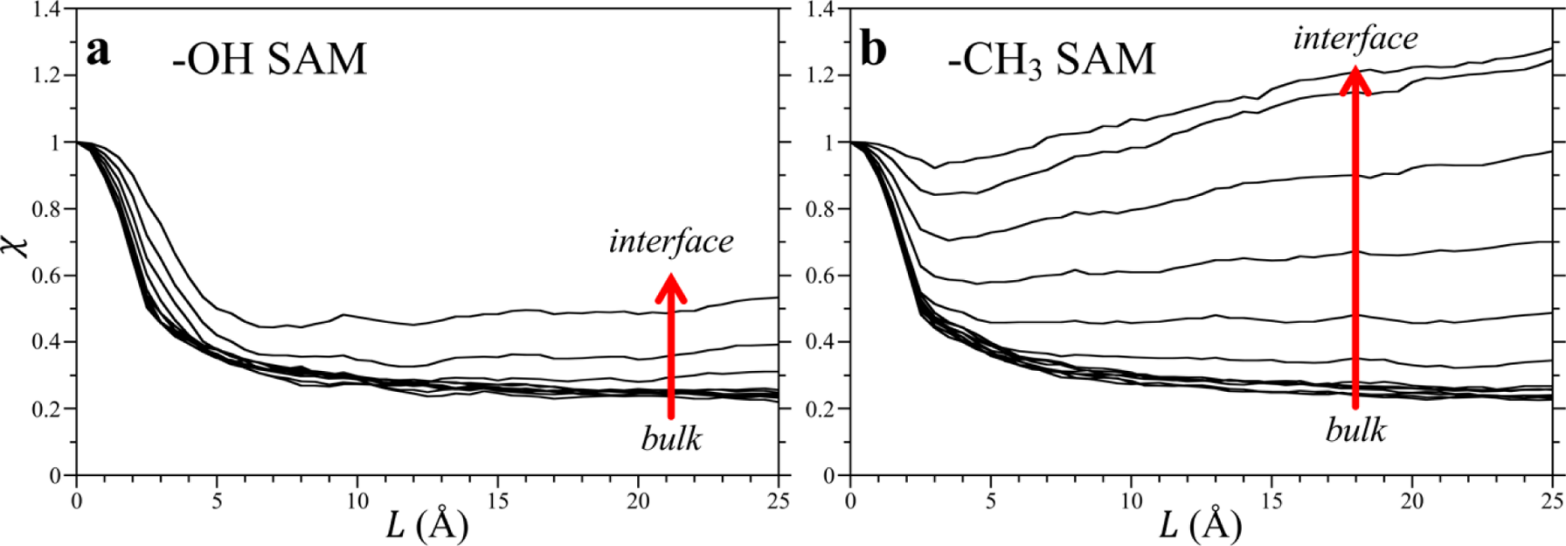
Normalized variance χ as a function of *L* for cuboidal observation volumes (*L* × *L* × 3 Å^3^) placed at different *z* locations from the −OH (left) and −CH_3_ (right) SAM surfaces in TIP3P water. As described in the caption of Figure 3, there are 11 in (a) and 14 curves in (b) spanning the region from the bulk to the interface.

The normalized variance, χ, in cuboidal volumes with dimensions of *L* × *L* × 3 Å^3^ next to SAM surfaces are shown in Figure 4 as a function of the length *L* of the cuboid. We maintained the width to be constant, equal to 3 Å, which is approximately equal to the size of a water molecule. The choice of such a thin cuboid as an observation volume allows for calculations of water occupancy fluctuations near or farther away from the SAM surface, thereby providing local and bulk measurements of these quantities. The normalized variance depends on the size and shape of the volume of interest even in bulk water and therefore a direct comparison between the numerical values of χ for a cuboid and a sphere is not useful. Indeed, while the large radius limit for the spherical volume in the bulk liquid corresponds to the bulk compressibility, in the case of the cuboidal volume with a single fixed dimension (e.g., 3 Å in the *z* direction), the effective compressibility in the infinite volume limit can be distinct from the bulk compressibility. Nevertheless, it is instructive to compare how χ depends on the size of the observation volume in these inhomogeneous environments.

For cuboids placed more than 20 Å or more away from either −CH_3_ or −OH SAM surfaces (Figure 4), that is, when the cuboid is effectively in bulk water, the variation of χ with *L* is qualitatively similar to that observed for a sphere in bulk water. Namely, χ(*L* =0) is one and subsequently decreases with increasing *L*, asymptotically approaching an infinite volume limit. As noted above, this asymptotic limit is not necessarily *k*_B_*T*ρ_w_κ_T_ because the width of the cuboid is not infinity but roughly the size of a water molecule. Rather, this effective or local compressibility can be thought of as a susceptibility of the water density within the observation volume to respond to external forces applied to that volume.^53^

Probing the cuboidal volumes approaching the SAM surface, however, both the variance and average of the water occupancy distribution are expected to be influenced by the surface. For cuboids placed 1, 1.5, and 2 Å from the origin (which is defined by the peak of the −OH head group density peak), although the qualitative behavior is χ *versusL* is similar to that in the bulk, the normalized variance is enhanced relative to the bulk. This does not indicate enhanced density fluctuations but is a result of the complex interplay between reduction in the density in the denominator and the bulk water-like correlations between water molecules in the cuboid mediated by the crystalline templating of the surface −OH group. In contrast, next to the −CH_3_-terminated SAM, χ exhibits a significantly different dependence on *L*. Namely, for cuboids placed in contact with the hydrophobic surface (at distances of 1, 1.5, 2, and 2.5 Å) fluctuations are greatly enhanced with no clear convergence of χ with increasing *L*. The values of χ for a cuboid of length 25 Å are many times greater than that in the bulk solution. These observations suggest that an extended hydrophobic surface has a more profound impact on density fluctuations, causing greater consternation in the waters in contact with the surface. These enhanced fluctuations are attributable to long-range capillary fluctuations at surfaces that help moderate large-scale hydrophobic interactions.^54,55^

The qualitative similarity of the dependence of water occupation fluctuations on size in cuboidal and spherical observation volumes in the bulk suggests that an interpolative fluctuation formula may be constructed for non-spherical volumes to bridge between the microscopic and macroscopic limits, although the infinite volume-normalized variance may depend on the details of the specific geometry. In this case, the asymptotic value in the bulk may be determined following eq 7, utilizing the appropriate integration domain. As hydrophobic interfaces are approached, however, it will be necessary to account for the large-scale fluctuations that are quenched at hydrophilic surfaces.

### Interpolated Gaussian Fluctuation Theory Predictions of the Cavity Solvation in Bulk Water

As laid out above, IGFT has the potential to predict the hydration properties of atomic-sized hard cavities in water. The inputs to the theory are the density, compressibility, and effective diameter of water (e.g., Figure 1), which enable the prediction of χ over a broad range of temperatures and solute sizes (Figure 2). Here, we assess the accuracy of IGFT at reproducing the unique hydration thermodynamics of non-polar species in water.

**Figure 5:**
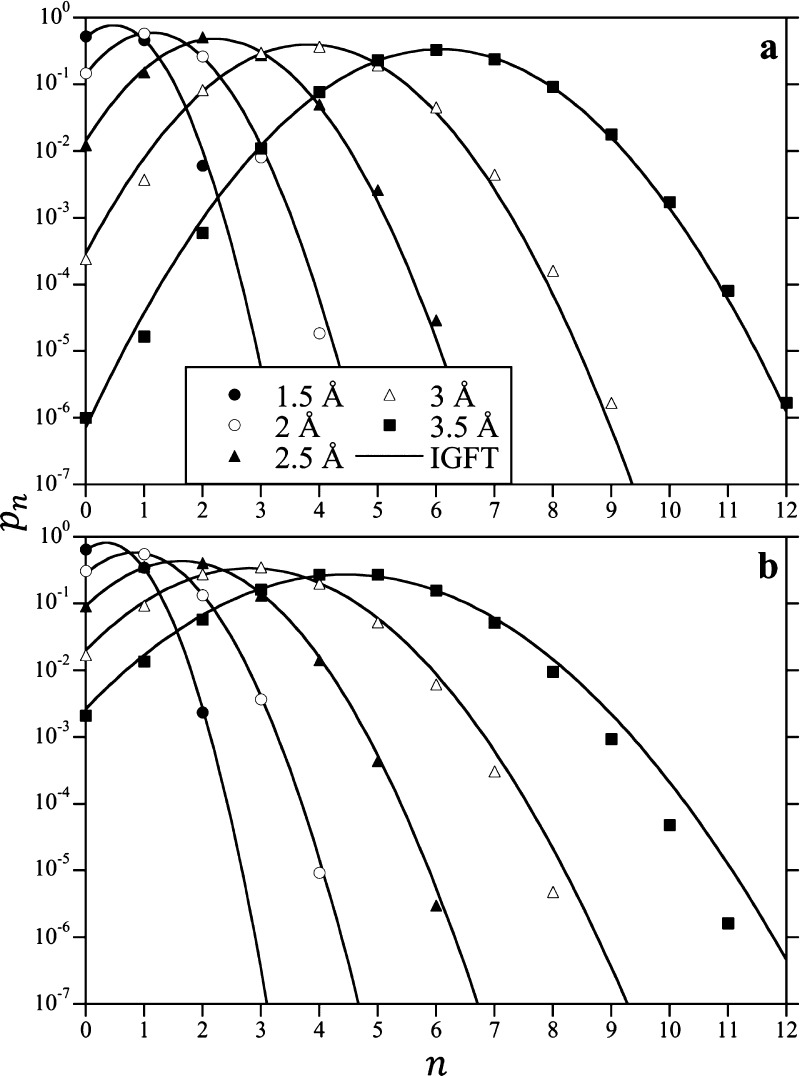
Cavity occupation probability distributions, *p*_*n*_, in water, as observed from the simulation and predicted by the IGFT and information theory at 300 bar at 25 (a) and 300 °C (b). The figure symbols for cavities with the solvent-excluded radii of 1.5, 2, 2.5, 3, and 3.5 Å are defined in the legend in (a). Simulation error bars are smaller than the line thicknesses.

The cavity water occupation probabilities as a function of *n* at 25 and 300 °C as determined from the simulation for observation volumes with radii of 1.5, 2, 2.5, 3, and 3.5 Å are compared with the predictions of IGFT in Figure 5. The probabilities obtained from the simulations are effectively parabolic on a log scale, that is, they are Gaussian. The predictions of IGFT are in excellent quantitative agreement with simulation values, especially for the case of an empty cavity (*n* = 0), although notable deviations are observed. At 25 °C, the probability of observing a single water molecule (*n* = 1) in a spherical volume with R = 3 and 3.5 Å is lower than that predicted by IGFT (Figure 5a). This behavior has been previously noted and ascribed to the formation of a vapor–liquid boundary layer about the solute for solvents near coexistence.^14^ For larger cavities, this tendency is reflected in the low-*n* fat tail observed in the solvent occupancy distribution, where the probability distribution is highly non-Gaussian especially for small values of *n*.^10^ This observation foretells that while IGFT accurately captures the occupation distribution for the cavities considered, it will break down as their size increases. At 300 °C, on the other hand, IGFT appears to over predict the occupation probabilities for *n* values larger than ⟨*n*⟩ for the R = 3 and 3.5 Å. We ascribe this deviation to the fact that IGFT slightly overpredicts the width of the Gaussian distribution with increasing temperature (Figure 2).

**Figure 6:**
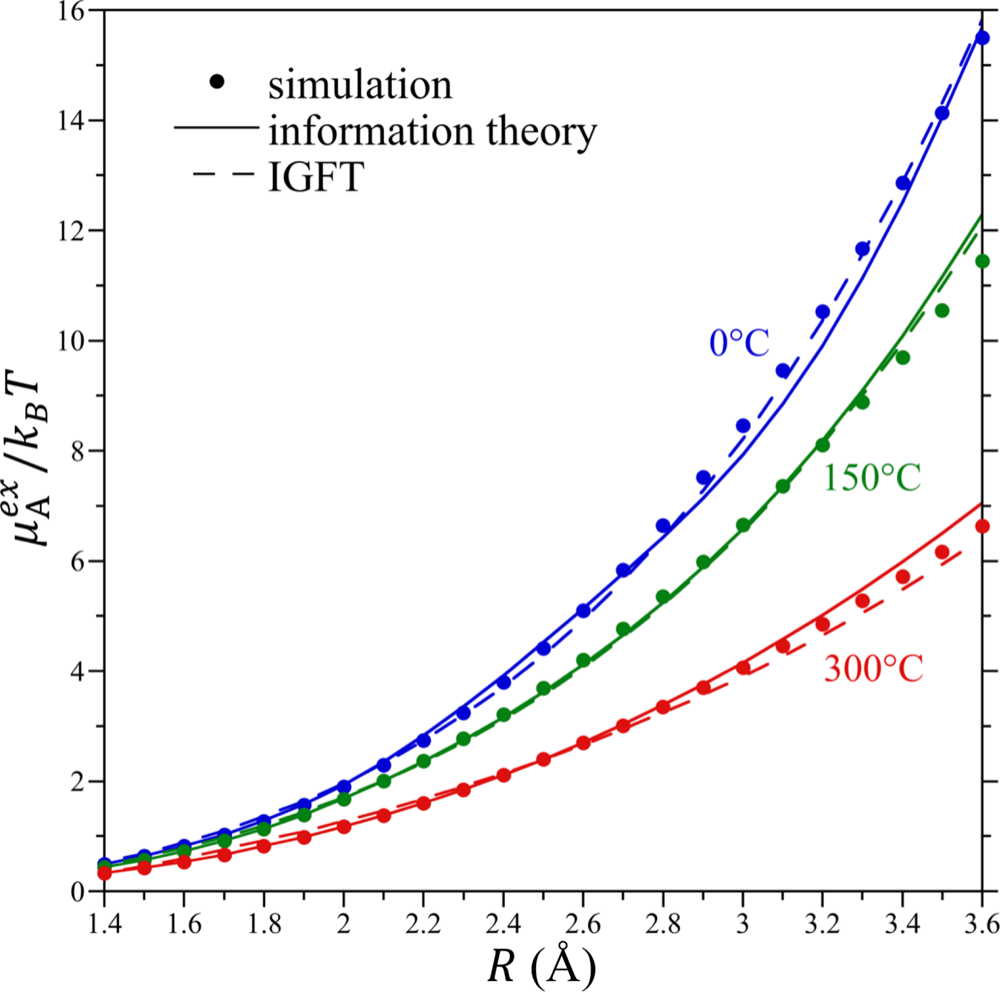
Hard-sphere solute excess chemical potential divided by *k*_B_*T* as a function of the solute radius from 1.4 to 3.6 Å at 0, 150, and 300 °C at 300 bar. Simulation results, predictions of the information theory, and IGFT predictions are reported in this figure. Symbols are as defined in the figure legend. Simulation error bars are smaller than the figure symbols.

The size dependence of the hydration free energies of HS solutes in water at 0, 150, and 300 °C as determined from the molecular simulation and IGFT are reported in Figure 6 over the range *R* = 1.4–3.6 Å. IGFT predicts the free energies of these solutes over the reported size range at each temperature remarkably well. The greatest deviations are found to occur with increasing solute size, as might be expected because the Gaussian approximation breaks down with increasing solute size. Given that IGFT represents an approximate solution to the information theory expression for the probability distribution (eq 2) subject to simulation constraints on the first and second moments of the cavity occupation distribution, it is worthwhile to compare information theory’s predictions for the HS solute chemical potentials against IGFT (Figure 6). While not perfect, IGFT closely tracks the predictions of the information theory and indeed appears to accurately reproduce the simulation results. This result is all the more remarkable given that the information theory contains information on the pair correlations between water molecules, as embodied in ⟨*n*^2^⟩, while IGFT neglects this information beyond the inclusion of the bulk compressibility.

**Figure 7:**
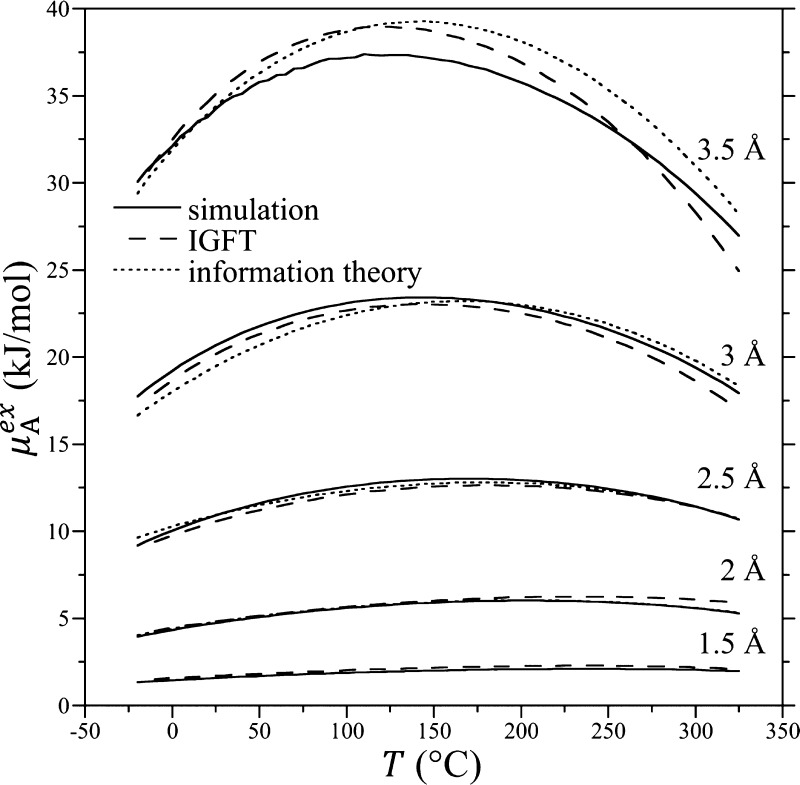
Solute cavity excess chemical potentials as a function of temperature at 300 bar for cavities of radii of 1.5, 2, 2.5, 3, and 3.5 Å (sizes identified in figure). Simulation, IGFT, and information theory results are reported. Symbols are as defined in the figure legend. Simulation error bars are smaller than or comparable to the thickness of the lines used.

Simulation results, IGFT, and information theory predictions for the excess chemical potentials of HS solutes with radii of 1.5–3.5 Å as a function of temperature are reported in Figure 7. The free energies increase with increasing temperature before passing through a maximum at elevated temperatures above the normal boiling point of water. The negative concavity of these curves is indicative of the large positive heat capacity increment associated with non-polar solute dissolution (i.e., because ∂^2^μ_A_^ex^/∂*T*^2^|_*P*_ = −*c*_A_^ex^/*T* < 0, it follows that *c*_A_^ex^ > 0, where *c*_A_^ex^ is the hydration heat capacity), while the initial positive slope in the free energy near room temperature indicates an unfavorable (i.e*.*, negative) hydration entropy, both of which are signatures of hydrophobic hydration. Overall, the agreement between the simulation and theoretical predictions is excellent, with a root-mean-square difference of 0.2 *k*_B_*T* over all solute sizes and a maximum difference of 0.4 *k*_B_*T* for the 3.5 Å solute. The largest errors in IGFT’s predictions are observed for the *R* = 3.5 Å solute, which is anticipated given that the Gaussian approximation breaks down for solutes of increasing size. Nevertheless, the theory accurately captures the temperature dependence of the free energy. The agreement between IGFT and information theory is nearly quantitative, although notable differences are observed for the 3.5 Å solute. Nevertheless, for the 3.5 Å solute, the information theory has a root-mean-square difference with simulation of 0.4 *k*_B_*T*, comparable in accuracy with the IGFT. Tracing the difference between the IGFT and information theory predictions, we find that the difference does not arise from the assumption that the occupation fluctuations are continuous (eq 4) instead of discrete (eq 2). Rather, the difference arises from the interpolation formula used by the IGFT for χ (eq 13), as opposed to using the occupation distribution moments evaluated from the simulation, as used by the information theory. The lower free energy predicted by IGFT with increasing temperature compared to the information theory can be directly traced to IGFT’s prediction of a greater variance than observed from the simulation as the critical point is approached (Figure 2).

The enthalpy, entropy, and heat capacity of hydrophobic hydration can be determined by fitting the temperature-dependent simulation results and theoretical predictions to the expression
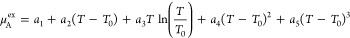
15where *T*_0_ is a reference temperature, taken here to be 298.15 K (25 °C), and the *a*_*i*_’s are fitted constants. The form of this expression assumes the hydration heat capacity has a parabolic dependence on the temperature (i.e., *c*_A_^ex^ = −*T*∂^2^μ_A_^ex^/∂*T*^2^|_*P*_ = −*a*_3_ – 2*a*_4_*T* – 6*a*_5_*T*(*T* – *T*_0_)), which is reasonable for fitting over a wide temperature range given that the hydration heat capacity is expected to be a decreasing function of temperature at lower temperatures and an increasing function of temperature as the critical point is approached.^56−59^ The hydration enthalpy (*h*_A_^ex^ = −*T*^2^∂(μ_A_^ex^/*T*)/∂*T*|_*P*_) and entropy (*s*_A_^ex^ = −∂μ_A_^ex^/∂*T*|_*P*_) similarly follow from appropriate temperature derivatives of eq 15.

**Figure 8:**
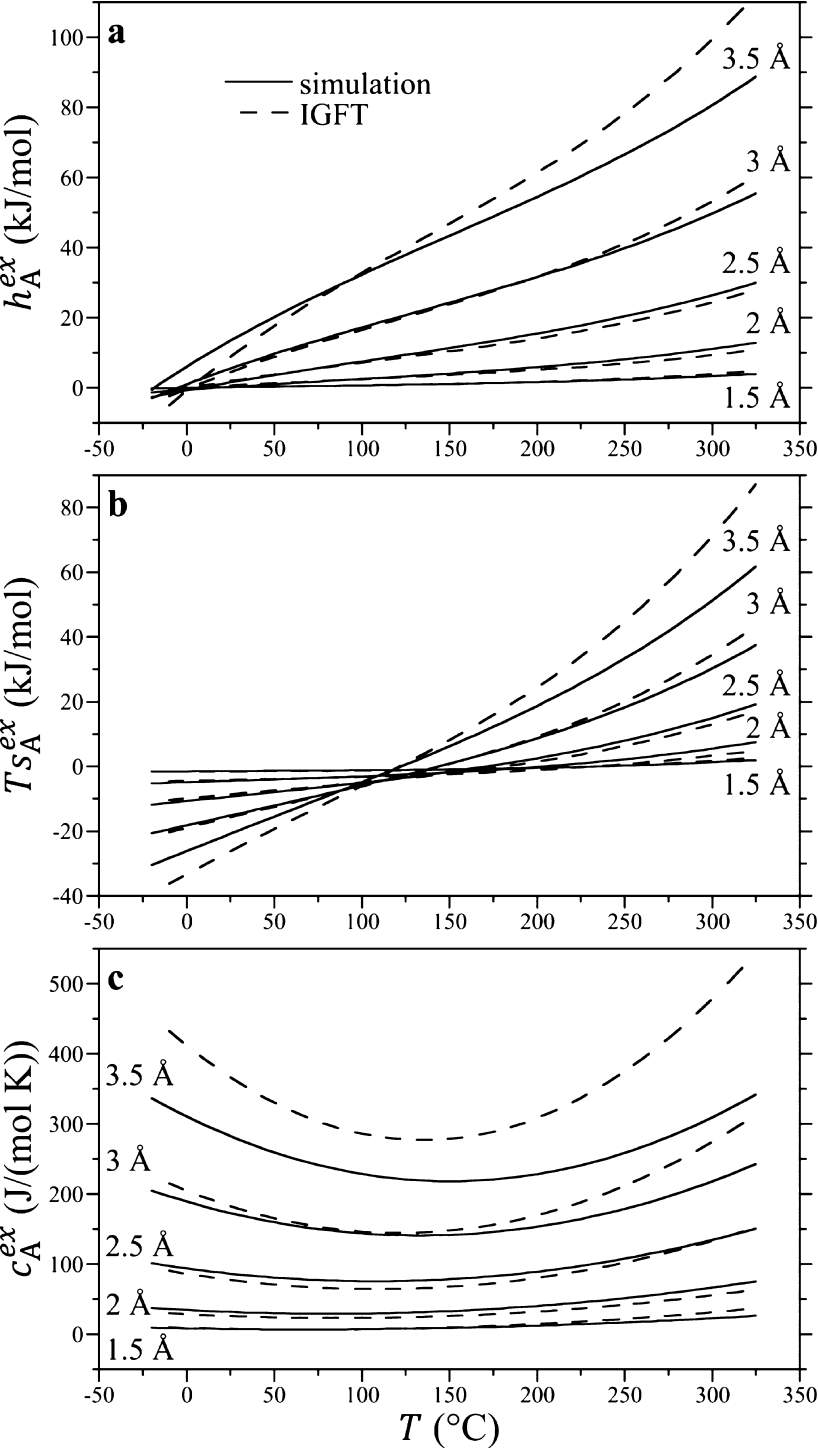
Hydration enthalpies (a), entropies (b), and heat capacities (c) as a function of temperature at 300 bar for cavities of radii of 1.5, 2, 2.5, 3, and 3.5 Å (sizes identified in figure). Both simulation results and theoretical descriptions are reported. The lines are defined in the figure legend in (a).

The hydration enthalpies determined from the simulation and IGFT for HS solutes 1.5–3.5 Å in radius are reported in Figure 8a. Overall, the hydration enthalpies are increasing functions of temperature that are initially negative at the lowest temperatures examined and become positive near the normal freezing point of water. These observations are consistent with a large positive hydration heat capacity increment (*c*_A_^ex^ = ∂*h*_A_^ex^/∂*T*|_*P*_). The temperature at which the enthalpy is zero reflects the point the solubility of the HS solutes is a minimum as determined by
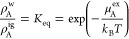
16where ρ_A_^w^ and ρ_A_^ig^ are the concentration/number density of the solute in the aqueous and ideal gas phases, respectively, and *K*_eq_ is the Ostwald solubility coefficient. The solubility minimum of HS solutes near water’s freezing point falls well below that observed for solutes such as methane, which experimentally occurs at 80 °C.^60^ This difference reflects the neglect of van der Waals interactions in the HS model, which shifts the solubility minimum up from the freezing point to more realistic temperatures.^19,61^ IGFT provides an excellent prediction of the simulation enthalpies over the entire temperature range considered. The largest differences are observed for the 3.5 Å solute at elevated temperatures. Notably, all the predicted enthalpies also appear to cross zero at slightly different temperatures than from simulation, although they all fall in a similar temperature range. These differences are discussed in more detail below.

The product of the temperature and hydration entropies determined from the simulation and IGFT for HS solutes 1.5–3.5 Å in radius are reported in Figure 8b. As with the enthalpy, the entropies are increasing functions of temperature as a result of their positive hydration heat capacities (*c*_A_^ex^ = *T*∂*s*_A_^ex^/∂*T*|_*P*_). Below 100 °C, all of the hydration entropies are negative, indicative of cavity solute hydration being entropically unfavorable. With increasing temperature, however, the entropies ultimately become positive. Over the temperature range 100–200 °C, the entropies of all the solutes examined appear to cross one another. The temperature at which the entropies of any two solutes are equal is referred to as their entropy convergence temperature.^7,19,62−66^ IGFT accurately reproduces the hydration entropies of all the solutes, although more significant, positive differences are observed for the 3.5 Å solute at elevated temperatures. The positive differences in the entropy appear to compensate for the positive differences in the enthalpy for the 3.5 Å solute (Figure 8a), giving rise to a reasonable prediction for the excess chemical potential of this solute over the entire temperature range (Figure 7).

The hydration heat capacities determined from the simulation and IGFT for HS solutes of 1.5–3.5 Å radius are reported in Figure 8c. As anticipated above, these heat capacities are large and positive. Moreover, they exhibit a non-monotonic temperature dependence as observed experimentally,^56−59^ passing through a minimum above the normal boiling point of water. The agreement between the simulation and IGFT is excellent for solutes of 3 Å radius and smaller. In the case of the 3.5 Å solute, IGFT predicts heat capacities that are greater than that observed from the simulation by about 35% on average. This difference is reflected in the more significant temperature dependence of the enthalpy and entropy observed for this solute (Figure 8a,b).

**Figure 9:**
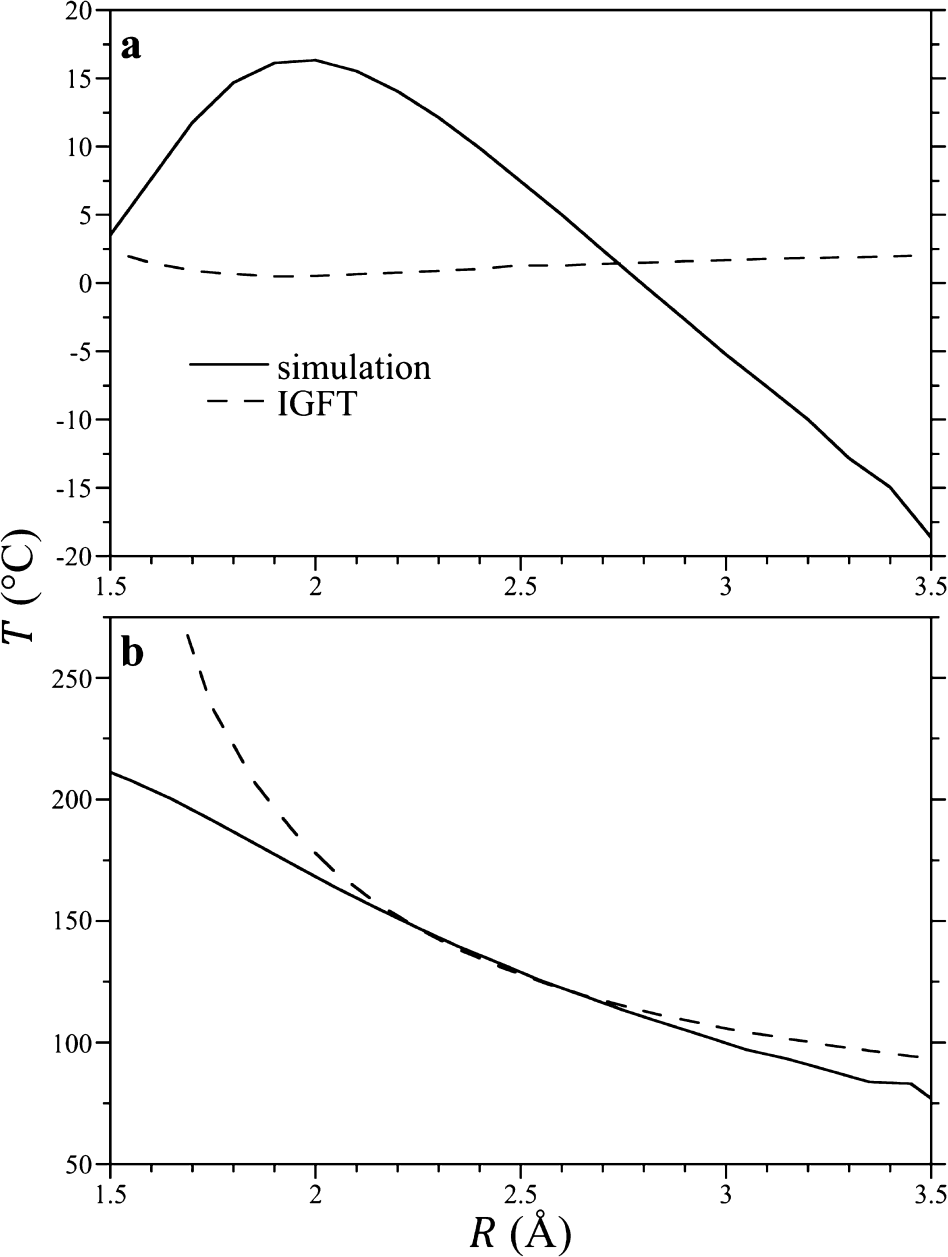
Solute solubility minima and entropy convergence temperatures as a function of the solute cavity radius, as determined from the simulation and theory. Figures (a,b) report the solubility minimum temperature (determined as *h*_A_^ex^ = 0) and the entropy convergence temperature (determined as ∂*s*_A_^ex^/∂*R* = 0), respectively. The lines are defined in the figure legend in (a).

As noted above, the temperature at which the hydration enthalpy is zero (*h*_A_^ex^ = 0) corresponds to the point at which the Ostwald solubility, embodied in *K*_eq_, is a minimum. In Figure 9a, we compare the IGFT predictions of the solubility minimum temperature as a function of the solute size against simulation results for the HS solutes. While the simulation results and IGFT predictions appear to converge to one another as the solute size gets smaller, IGFT fails to predict the non-monotonic dependence of the solubility minimum temperature as a function of the solute size determined from the simulation. Specifically, our simulations find the solubility minimum appears to increase from temperatures close to freezing for the smallest solute examined (*R* = 1.5 Å) to a maximum near a solute ∼2 Å in radius. After this point, the solubility minimum temperature falls with the increasing solute size, ultimately dropping below the freezing point of water for solute radii larger than ∼2.7 Å. Above the solubility minimum temperature, the hydration enthalpy is positive and opposes dissolution. Given that the enthalpy for creating an air/water interface is similarly positive over all temperatures, it might not be surprising to find that the solubility minimum temperature drops below the freezing point of water with increasing solute size, although it is surprising to find this signature for interface formation occurring for such small solutes. This should be coupled, however, with the observation that the hydration entropy (Figure 8b) is also negative at 0 °C. The entropy of forming a macroscopic interface is positive, on the other hand, so that the surface tension is a decreasing function of temperature.

A second behavior of interest is the observation that the entropies of solutes of different sizes cross one another at a so-called entropy convergence temperature. This is not a unique temperature for HS solutes but is distinct for each potential pair of solutes. The convergence temperature can be more practically defined as the temperature at which the excess hydration entropy of a given solute and one differentially larger are equal^19,63^
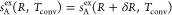
17This condition is satisfied when ∂*s*_A_^ex^/∂*R*|_*P*_ = 0. The entropy convergence temperatures as a function of *R* determined from the simulation and IGFT are reported in Figure 9b. In both cases, the convergence temperatures are decreasing functions of the solute size. For solutes 2–3 Å in radius, the simulation and IGFT predictions appear to converge to one another, dropping over this range from 150 to 100 °C. For solutes larger than 3 Å, the simulations and theory appear to diverge from one another, although the difference between the two is no larger than 12 °C up to 3.5 Å. Below 2 Å, however, the IGFT predictions rise outside the bounds of the temperatures simulated, while the simulation results are well behaved. Indeed, following the exact results that the chemical potential is μ_A_^ex^ = −*k*_B_*T*ln(1 – 4π*R*^3^ρ_w_/3) for *R* < *d*_ww_/2, the convergence temperature for a solute with *R* = 0 occurs when *T*α = 1, where α is the thermal expansion coefficient of the solvent. For TIP4P/2005 at 300 bar, we find *T*_conv_(*R* = 0) = 268 °C, which is a reasonable extrapolation for the simulation results shown in Figure 9b. We ascribe the unphysically large rise in the convergence temperature predicted by IGFT as *R* decreases to the theory’s incorrect treatment of the hydration free energy of sub-point-like solutes. Nevertheless, real atomic solutes such as helium through xenon have effective solvent-excluded radii in the range of 2.7–3.5 Å, for which IGFT does an excellent job.

## Conclusions

Here, we constructed an analytical approximation to describe the variance in the occupancy of water within a spherical observation volume within the liquid. This approximation is based on the known functional form of the variance for microscopically small volumes and the macroscopic limit determined by the bulk solvent compressibility. We constructed a polynomial bridge that interpolates between these two limits and describes occupation fluctuations in volumes of intermediate size. When coupled with the information theory conclusion that the probability of observing *n* waters within the observation volume is effectively Gaussian, we were able to derive an analytical expression for the free energy of hydration of the hard-sphere atomic-sized solutes that relies only on the density, compressibility, and effective diameter of liquid water. The free energies derived from this interpolated Gaussian fluctuation theory were shown to accurately reproduce the free energies, enthalpies, entropies, and heat capacities determined from the simulation of solutes up to 3.5 Å in radius, comparable in size to xenon. Moreover, the interpolated Gaussian fluctuation theory does a reasonable job of describing solubility minima and entropy convergence temperatures, although it does not capture all the nuances of the simulation results for these higher-level effects. Nevertheless, the agreement between the simulation and theoretical results is remarkable given the simplicity of the information required by the theory to predict a wide range of signatures of hydrophobic hydration.

One take away from IGFT is that the distribution of atomically sized cavities that could host a non-polar solute does not necessitate information on the structure of liquid water beyond its effective diameter. This may be rather surprising given the frequently invoked idea that the water’s hydrogen bonding network is fortified by the dissolution of non-polar solutes, giving rise to unfavorable clathrate-like structures.^67^ IGFT, on the other hand, does not speak to this structural stabilization despite the fact that hints of these clathrate structures have been noted from both the experiment^68−70^ and simulation.^71−74^ We may ask then, what role might these compelling structures play in the non-polar hydration process? Given that non-polar solute hydration is well described by a Gaussian theory, we may conclude any potential structuring of water about atomic-scale non-polar solutes is explored over the course of their ambient liquid-state fluctuations.^75^ Given that these fall within the bounds of Gaussian density fluctuations suggests that the work to form them is not onerous, as it would be in the case of a much larger cavity fluctuation in which the hydrating waters begin to resemble a macroscopic interface. The fact that the knowledge of water’s equation-of-state is sufficient to describe these Gaussian fluctuations suggests that the spontaneous formation of structures about voids in water is a constituent of the equation-of-state itself and not a direct consequence of the introduction of an actual solute into solution.

IGFT is not limited to spherical solutes, although the details of the interpolating polynomial are geometry dependent. Specifically, the limiting second derivative of χ being zero (eq 11b) is a consequence of the integration domain of the χ integral being spherical (eq 8). Application of the theory to alternate geometries would subsequently necessitate the inclusion of the second-order term in eq 12, which could have consequences on the application of the theory beyond spherical geometries. In addition, while the theory does an excellent job at describing the hydration of individual solutes, we do not expect it to be able to describe hydrophobic interactions between solute pairs because the packing of water certainly plays a role in the oscillations in the potentials-of-mean force between solutes. Notably, IGFT cannot predict features such as the solvent-separated minimum observed in the potential-of-mean force between methanes in water due to the neglect of solvent correlations.^6^ The observation here that the normalized density fluctuations within cuboidal volumes is qualitatively similar to those found in spherical volumes supports the potential for extending IGFT to diverse shapes, although the variance of those fluctuations near distinct surfaces highlights the challenges in extending the theory to predict interactions.

Finally, it is of interest to examine the application of IGFT to non-aqueous solvents or coarse-grained models of water and other solvents.^76^ We expect the greatest utility will be for effectively monoatomic solvents such as water (its hydrogens are typically neglected when considering non-polar solute hydration). Intramolecular bonding in polymers, for instance, introduces significant deviations from Gaussian behavior, although the lumping of groups can alleviate this difficulty.^77^
